# Terahertz Wave Alleviates Comorbidity Anxiety in Pain by Reducing the Binding Capacity of Nanostructured Glutamate Molecules to GluA2

**DOI:** 10.34133/research.0535

**Published:** 2024-12-11

**Authors:** Zihua Song, Yuankun Sun, Pan Liu, Hao Ruan, Yuanyuan He, Junkai Yin, Chun Xiao, Jing Ma, Yun Yu, Shaomeng Wang, Yubin Gong, Z. W. Lin, Zhi Zhang, Chao Chang, Maojun Yang

**Affiliations:** ^1^ Innovation Laboratory of Terahertz Biophysics, National Innovation Institute of Defense Technology, Beijing 100071, China.; ^2^ School of Electronic Science and Engineering, University of Electronic Science and Technology of China, Chengdu 611731, China.; ^3^ School of Safety Engineering, North China Institute of Science and Technology, Hebei 065201, China.; ^4^ School of Life Sciences, Tsinghua University, Beijing 100081, China.; ^5^ School of Life Science and Technology and Frontier Institute of Science and Technology, Xi’an Jiaotong University, Xi’an 710049, China.; ^6^ Division of Life Sciences and Medicine, Department of Anesthesiology, the First Affiliated Hospital of USTC, Hefei National Laboratory for Physical Sciences at the Microscale, University of Science and Technology of China, Hefei 230026, China.; ^7^ School of Physics, Peking University, Beijing 100081, China.

## Abstract

Comorbid anxiety in chronic pain is clinically common, with a comorbidity rate of over 50%. The main treatments are based on pharmacological, interventional, and implantable approaches, which have limited efficacy and carry a risk of side effects. Here, we report a terahertz (THz, 10^12^ Hz) wave stimulation (THS) technique, which exerts nonthermal, long-term modulatory effects on neuronal activity by reducing the binding between nano-sized glutamate molecules and GluA2, leading to the relief of pain and comorbid anxiety-like behaviors in mice. In mice with co-occurring anxiety and chronic pain induced by complete Freund’s adjuvant (CFA) injection, hyperactivity was observed in glutamatergic neurons in the anterior cingulate cortex (ACC^Glu^). Using whole-cell recording in ACC slices, we demonstrated that THS (34 THz) effectively inhibited the excitability of ACC^Glu^. Moreover, molecular dynamics simulations showed that THS reduced the number of hydrogen bonds bound between glutamate molecules and GluA2. Furthermore, THS target to the ACC in CFA-treatment mice suppressed ACC^Glu^ hyperactivity and, as a result, alleviated pain and anxiety-like behaviors. Consistently, inhibition of ACC^Glu^ hyperactivity by chemogenetics mimics THS-induced antinociceptive and antianxiety behavior. Together, our study provides evidence for THS as an intervention technique for modulating neuronal activity and a viable clinical treatment strategy for pain and comorbid anxiety.

## Introduction

Various brain stimulation techniques have been utilized to regulate brain functions and treat brain disorders, such as transcranial magnetic stimulation, deep brain stimulation, and transcranial direct-current stimulation, which have been employed to intervene in patients with pain [1–3]. However, these stimulation techniques may only provide relief for specific types of pain in certain individuals, and there is a lack of agreement on the effectiveness of clinical treatment recommendations and guidelines. The outcome of the procedure is influenced by various factors, such as the optimized stimulation parameters and dosage, as well as intracranial target selection. Terahertz (THz) waves have been extensively shown to have a wide range of effects on biological functions, including in bioimaging, cancer, ion channel and neural regulation studies [4–8]. The impact of THz waves on neuronal firing rates or potassium channel has been observed in mice sound-licking associative learning task [9] or pigeons’ eye movements [10]. Irradiation (34.5 THz) nonthermally restores in vivo cognitive function in rats with posttraumatic stress disorder by improving the expression of NR2B and phosphorylated NR2B [11]. Molecular dynamics simulations show that 42.55 THz enhances the interactions in Aβ42 monomers and dimers [12]. Irradiation of 13.02 THz can promote nicotine dissociation from acetylcholine-binding protein by disrupting hydrogen bonds [13]. Irradiation of 11.05 and 21.44 THz may increase nicotine aggregation, while 19.05 THz increases epinephrine aggregation [14]. Growing evidence supports that the effects of THz waves may shed some light on potential therapeutic strategies for diseases of the nervous system. These results indicate that certain characteristics of THz waves may play a critical role in the neurological impact of THz waves on pain perception. However, the mechanism of action of THz technology remains unclear, and the potential risk of tissue damage from overheating is still undetermined.

Chronic pain is often linked to both physical and mental issues, including anxiety [15,16]. The brain regions implicated in the chronic pain often overlap with those involved in regulating anxiety, such as the anterior cingulate cortex (ACC), medial prefrontal cortex, and amygdala [17–19]. Research has shown that the ACC serves a dual function in processing emotional reactions and the sensory components of pain [20,21]. Both rodents and human patients with chronic pain have shown increased activity in the ACC, while deactivation of the ACC leads to both anxiolytic and analgesic effects [22–24]. However, traditional analgesics are often ineffective in treating pain symptoms in anxiety patients [25], presenting a marked challenge in effectively treating concurrent anxiety and pain symptoms. Neuroimaging evidence indicates that the ACC is a crucial area for the central modulation of emotional pain [26,27]. This suggests that the ACC has substantial clinical relevance for the central sensitization of pain and the regulation of emotions. To date, it remains unclear how the THz wave stimulation (THS) regulates ACC neuronal activity and comorbid anxiety symptoms in pain.

The α-amino-3-hydroxy-5-methyl-4-isoxazole propionic acid receptors (AMPARs) are responsible for primarily facilitating excitatory synaptic communication in the central nervous system (CNS). AMPAR disorders, which cause an imbalance in excitatory/inhibitory synaptic transmission, have been linked to the development of conditions like pain, depression, epilepsy, and stroke and are also considered as effective regulatory targets [28,29]. AMPARs are made up of 4 subunits, GluA1, GluA2, GluA3, and GluA4, and are ligand-gated channels. The most common combination in the forebrain is the GluA1/GluA2 heterotetramer. GluA2 having a glutamate-binding pocket that plays a particularly important role in glutamate binding [30]. However, the precise molecule and the role of the AMPAR in mediating THS-induced pain relief are still not well understood.

**Fig. 1.**
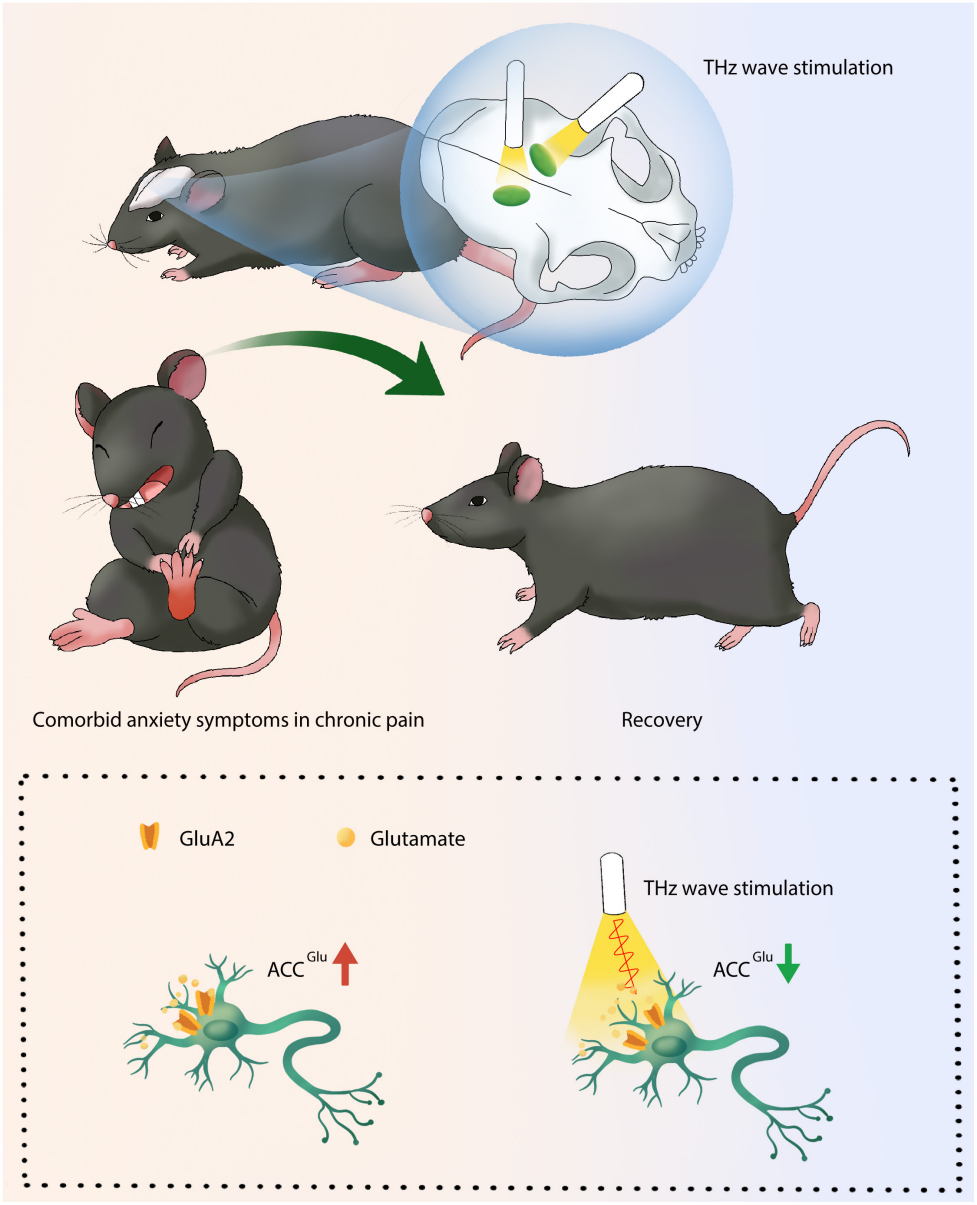
Thirty-four-terahertz wave promotes pain relief with anxiety-like behavior by reducing the ability of glutamate to bind to the GluA2 subunit. The excitability of ACC^Glu^ neurons is increased in CFA3D mice showing signs of nociceptive hypersensitivity combined with anxiety. Thirty-four-terahertz wave irradiation increases the vibration of glutamate with the GluA2 subunit, and this vibration decreases the binding capacity between glutamate and the GluA2 receptor, which attenuates ACC^Glu^ neuronal hyperactivity, leading to the relief of pain accompanied by anxiety-like behavior.

This study utilized THS and whole-cell clamp recording to show that THS decreased hyperactivity in glutamatergic neurons in the ACC (ACC^Glu^). This was achieved by acting on the arginine 96 (Arg96) of GluA2 and glutamate in the binding pocket, resulting in inhibition of the development of pain sensitization and anxiety-like behaviors in mice induced by complete Freund’s adjuvant (CFA) (Fig. 1). Chemical manipulation of ACC^Glu^ can recapitulate the THS-induced antinociceptive and antianxiety effects. Therefore, our study offers a feasible and practical framework for understanding the modulatory effects on neuronal signaling and behavior exerted by THS with a specific frequency. It also implicates potential therapeutic strategies for addressing anxiety in individuals with pain conditions.

## Results

### THS reduces ACC^Glu^ neuronal activity

**Fig. 2.**
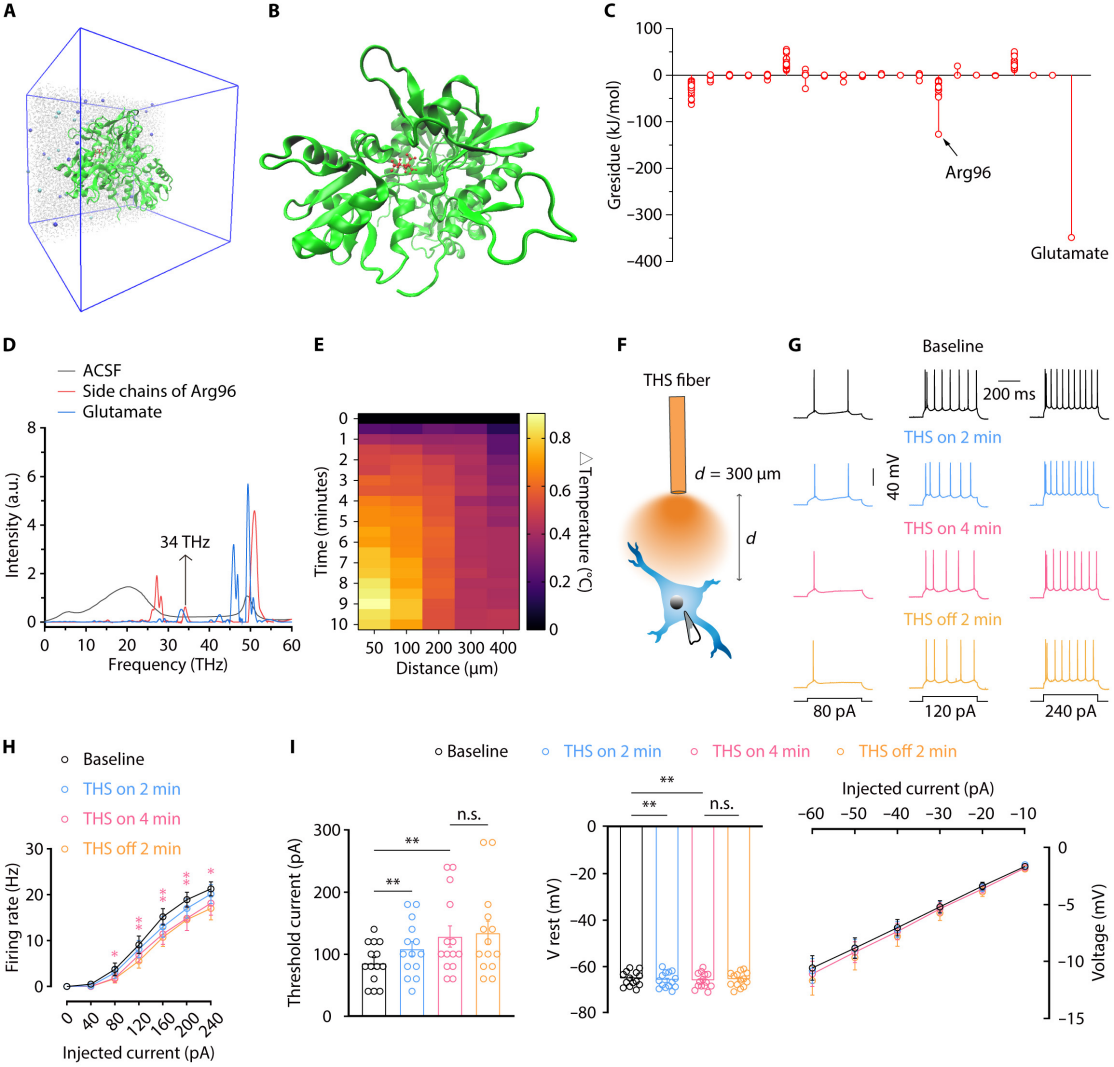
THS reduces ACC^Glu^ neuronal activity. (A and B) The complete simulation system consisting of the glutamate (red) and AMPAR (green) solvated in the NaCl solution (A) and glutamate-AMPAR complex (B). (C) Molecular dynamics simulation of residue contributions to the binding free energy in the base state. All residues are categorized to the type, and each circle denotes one residue. Glutamate represents the glutamate molecule. (D) The absorption spectra of the side chains of Arg96, glutamate molecule, and ACSF. (E) A heat map of temperature changes over time based on the distance between the THS fiber and the temperature sensor. (F) Schematic illustration of THS and whole-cell recording in the ACC slice. Representative traces (G) and summarized data (H) of action potentials. (I) Rheobase, voltage–current plots of voltage responses to a stepwise series of hyperpolarizing currents (−10 to −60 pA, −10 pA/step; duration: 500 ms), resting membrane potential, and input resistance recorded from glutamatergic neurons in the ACC slices from mice before, during, and after THS (*n* = 14 neurons from 3 mice). Data are shown as means ± SEM. *P < 0.05; **P < 0.01. n.s., not significant. One-way repeated-measures ANOVA with Tukey analysis was used for (H). A 2-tailed paired Student *t* test was used for (I).

GluA2 is an essential subunit of AMPARs, contains a binding pocket for glutamate binding to the AMPAR, mediates rapid excitatory communication throughout the CNS, and is critical for maintaining synaptic strength. Imbalance in AMPAR signaling has been linked to various neurological disorders [30]. To investigate the contribution of glutamate and various residues to the binding free energy of GluA2, we set up a complete molecular dynamics simulation system (Fig. 2A and B) and found that Arg96 and glutamate contributed the most to the binding free energy of glutamate and GluA2 (Fig. 2C). Prior research has demonstrated that 83 THz can increase the strength of the bond between histone and DNA [31]. To explore the absorption of THz waves in detail, molecular dynamics simulations were used to calculate the absorption spectrum of the side chains of Arg96 in the glutamate-binding pocket of GluA2. Our results showed that Arg96 exhibits a unique fingerprint peak near the frequency of 34 ± 1 THz, distinct from the strong absorption range of the artificial cerebrospinal fluid (ACSF) (Fig. 2D), indicating that approaching 34-THz waves mainly has a direct effect on the Arg96. We thus chose 34 THz with low attenuation of light in ACSF for our experimental study [32]. The high pulse energy of intense ultrabroadband laser sources is garnering increasing interest across various disciplines such as biology, chemistry, physics, and material science [33]. The coupler collimates the laser output from a quantum cascade laser (QCL) before it is coupled into the fiber. The fiber, which has a core diameter of 400 μm, provides flexibility in changing the stimulation target. The THS was delivered with the following parameters: the power density at the tip of the laser fiber in air was approximately 1 or 6 W/cm^2^, pulse width 500 ns, and pulse rate 200 kHz. Previous studies have shown that near-infrared laser irradiation has photothermal therapeutic effects on cells, and heating can influence the activity of neurons [34–36]. To avoid thermal effect and tissue damage, the temperature was kept below 2 °C [37]. To examine the temperature change resulting from THS, we conducted a time-dependent analysis of the thermal response of a fiber over a specific range of irradiation using the temperature sensor of the automatic thermostat (TC-344C). In a slice recording chamber filled with ACSF, we recorded the temperature changes at various distances between the 6-W/cm^2^ optical fiber tip and the sensor within 10 min. When applying THS, the maximum temperature 300 μm from the tip of the optical fiber increased by approximately 0.45 °C within 10 min and reached a steady state (Fig. 2E), which can be a negligible temperature change.

To investigate how THS impacts neuronal excitability, we conducted whole-cell current-clamp recording from ACC^Glu^ neurons before, during, and after THS. In order to avoid any impact from the rise in temperature, recordings were taken from neurons located 300 μm away from the tip of the optical fiber (Fig. 2F). After current injections, we observed a decrease in the number of spikes in the ACC^Glu^ during 6 W/cm^2^ THS for 4 min compared to before THS. Additionally, we noticed a rise in rheobase, accompanied by a reduction in membrane input resistance. This inhibitory effect persisted for at least 2 min following cessation of irradiation (Fig. 2G to I). However, no notable alterations were observed in the membrane input resistance during 6 W/cm^2^ THS compared to before and after 6 W/cm^2^ THS (Fig. 2I). Interestingly, we found that 1 W/cm^2^ THS did not have a substantial impact on the excitability of ACC^Glu^ neurons (Fig. S1). Together, these findings indicate a nonthermal effect of 6 W/cm^2^ THS on neuronal excitability and responsiveness.

### Increased ACC^Glu^ neuronal excitability contributes to comorbidity of pain and anxiety-like behavior in mice

**Fig. 3.**
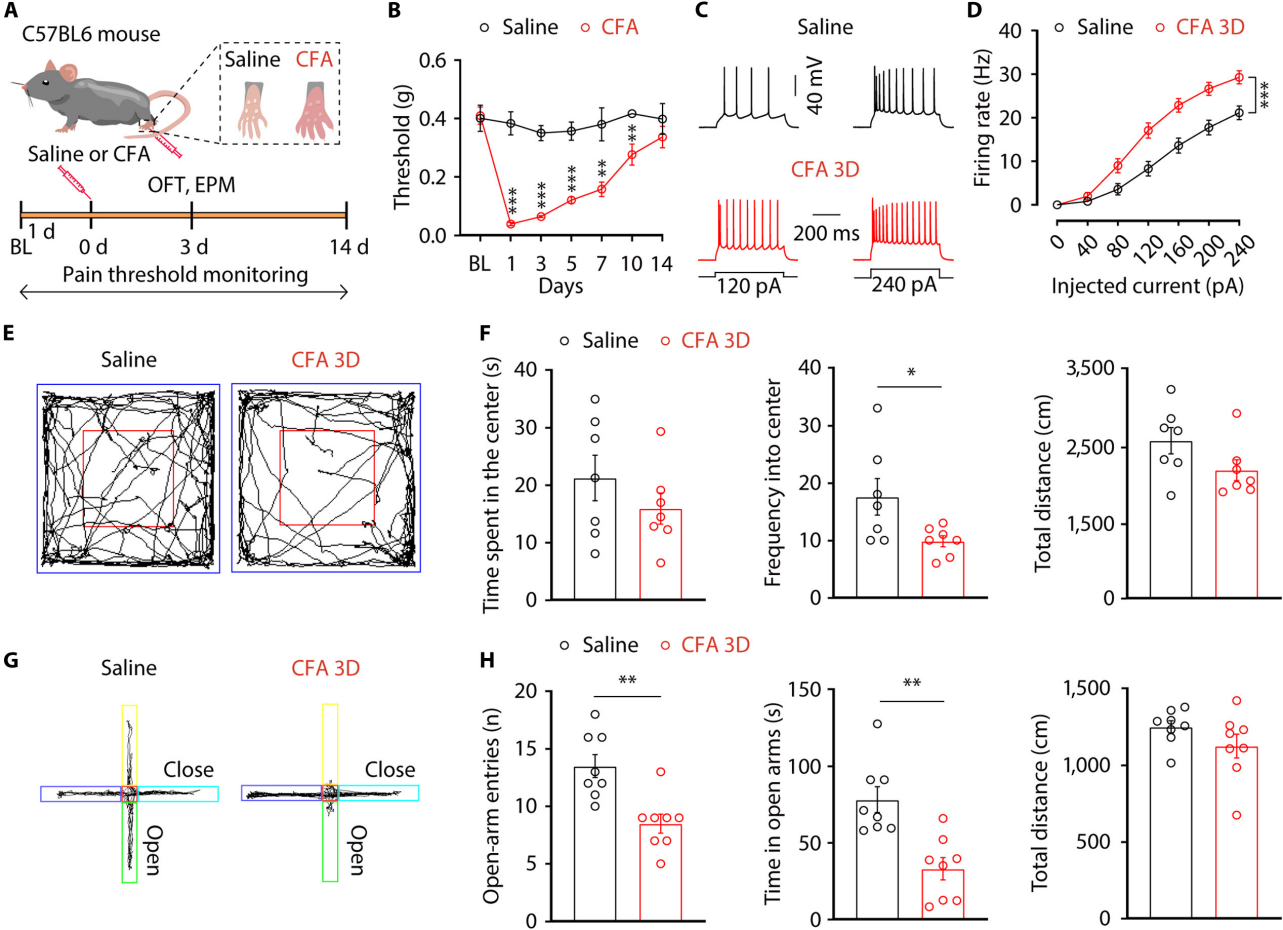
Increased ACC^Glu^ neuronal excitability in comorbid anxiety symptoms in CFA 3D pain mice. (A) Outline of the experimental procedure for CFA treatment and behavioral tests. (B) Time course of CFA-induced sensory pain (*n* = 8 mice per group). Representative traces (C) and summarized data (D) of action potentials recorded from glutamate neurons in the ACC slices from CFA 3D and saline mice (*n* = 21 to 22 neurons from 3 mice per group). (E) Representative animal tracks from mice treated with saline and CFA for 3 d in the OFT. (F) Performance of mice treated with saline or CFA 3D in the OFT (*n* = 7 mice per group). (G) Representative animal tracks from mice treated with saline and CFA for 3 d in the EPMT. (H) Performance of mice treated with saline or CFA 3D in the EPMT (*n* = 8 mice per group). Data are shown as means ± SEM. *P < 0.05; **P < 0.01; ***P < 0.001. Two-way repeated-measures ANOVA with Bonferroni post hoc analysis was used for (B) and (D). A 2-tailed unpaired Student *t* test was used for (F) and (H). BL, baseline.

Mice were used to establish comorbid anxiety symptoms in a pain model by inducing persistent inflammatory pain with CFA (Fig. 3A). Following CFA injection, the mice exhibited substantial tactile allodynia that could last for at least 10 d (Fig. 3B). Behavioral and neuronal activity tests were conducted on the third day after CFA injection (CFA 3D), during the process of pain development. We first determined the neuronal excitability in the ACC, a region known for its involvement in processing both internal and external sensory stimuli, as well as in the transformation of pain signals [38,39]. To study the functional role of ACC^Glu^ neurons in pain behavior regulation, whole-cell recordings were conducted on ACC^Glu^ neurons. We observed an increase in the spike firing rate in response to current injections (Fig. 3C and D) in ACC^Glu^ neurons of CFA 3D mice relative to saline mice. These results indicate heightened excitability of ACC^Glu^ neurons in mice experiencing pain.

Chronic pain is often accompanied by a series of psychological and physiological maladjustments, including anxiety [40]. To investigate whether the CFA-induced pain is associated with anxiety-like behaviors, we conducted corresponding behavioral tests on mice. The mice exhibited substantial anxiety-like behaviors in the routine assays, as shown in the open-field test (OFT), where CFA 3D mice reduced the frequency into center area compared to the control mice (Fig. 3E and F). Additionally, CFA 3D mice exhibited a reduction in both total entries and time spent in the open arms compared to the saline mice during the elevated plus maze test (EPMT) (Fig. 3G and H). Notably, there was no notable difference in the overall distance traveled by CFA 3D mice compared to saline mice during these tests (Fig. 3F and H). These findings indicate that anxiety-like behaviors are consistently triggered by the existing inflammatory pain model and that the activity of ACC^Glu^ neurons is heightened in this model.

**Fig. 4.**
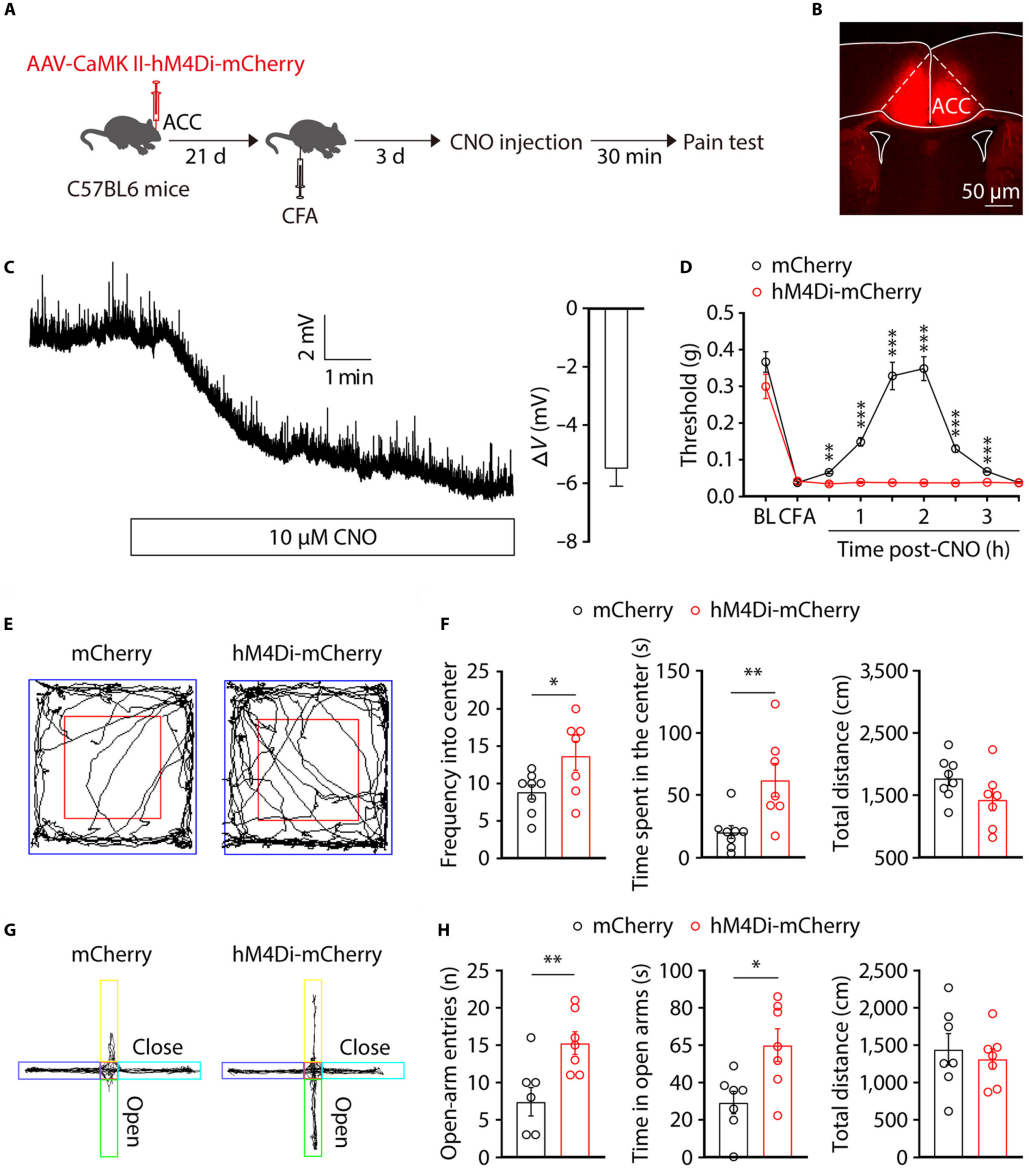
Chemogenetic inhibition of ACC^Glu^ neuronal activity alleviates pain sensitization and anxiety symptoms in CFA mice. (A) An outline of the experimental procedure for STZ mice with chemogenetic manipulations and behavioral tests. (B) Representative images showing the bilateral rAAV-CaMKIIa-hM4Di-mCherry (hM4Di) injection site within the ACC. Scale bar, 50 μm. (C) A representative trace (left) from a whole-cell recording from acute slices showing the effects of CNO on hM4Di-expressing neurons and statistics (right) showing the average magnitude of hyperpolarization (*n* = 5 neurons). (D) Effect of chemical genetic inhibition of ACC^Glu^ neurons on mechanical nociceptive thresholds of CFA 3D mice (*n* = 8 mice per group). (E) Representative animal tracks from CFA 3D mice after ACC infusion with AAV-DIO-mCherry or AAV-CaMKIIa-hM4Di-mCherry treated with CNO in the OFT. (F) Performance of CFA 3D mice after ACC infusion with AAV-CaMKIIa-mCherry or AAV-CaMKIIa-hM4Di-mCherry treated with CNO in the OFT (*n* = 7 to 8 mice per group). (G) Representative animal tracks from CFA 3D mice after ACC infusion with AAV-CaMKIIa-mCherry or AAV-CaMKIIa-hM4Di-mCherry treated with CNO in the EPMT. (H) Performance of CFA 3D mice after ACC infusion with AAV-CaMKIIa-mCherry or AAV-CaMKIIa-hM4Di-mCherry treated with CNO in the EPMT (*n* = 6 to 7 mice per group). Data are shown as means ± SEM. *P < 0.05; **P < 0.01; ***P < 0.001. A 2-tailed unpaired Student *t* test was used for (F) and (H). Two-way repeated-measures ANOVA with Bonferroni post hoc analysis was used for (D).

Based on the findings that increased activity of ACC^Glu^ neurons has a specific impact on pain and comorbid anxiety-like behaviors, we next investigated whether inhibiting these neurons could alleviate pain sensitization and anxiety in mice with CFA. Indeed, the chemogenetic inhibitory hM4Di expressing the CaMKIIa promoter was administered via injection into the ACC of mice (Fig. 4A to C). We then investigated whether inhibition ACC^Glu^ neurons could restore the pain sensitization and anxiety-like behaviors. The administration of the hM4Di ligand clozapine-N-oxide (CNO) via intraperitoneal injection successfully reversed the pain sensitization and anxiety-like behavior induced by CFA in mice (Fig. 4D to H). Taken together, these results indicate that suppressing ACC^Glu^ neurons can alleviate both the allodynia and the anxiety-like behavior in CFA 3D mice.

### THS decreases the excitability of ACC^Glu^ neurons and relieves pain accompanied by anxiety-like behaviors

**Fig. 5.**
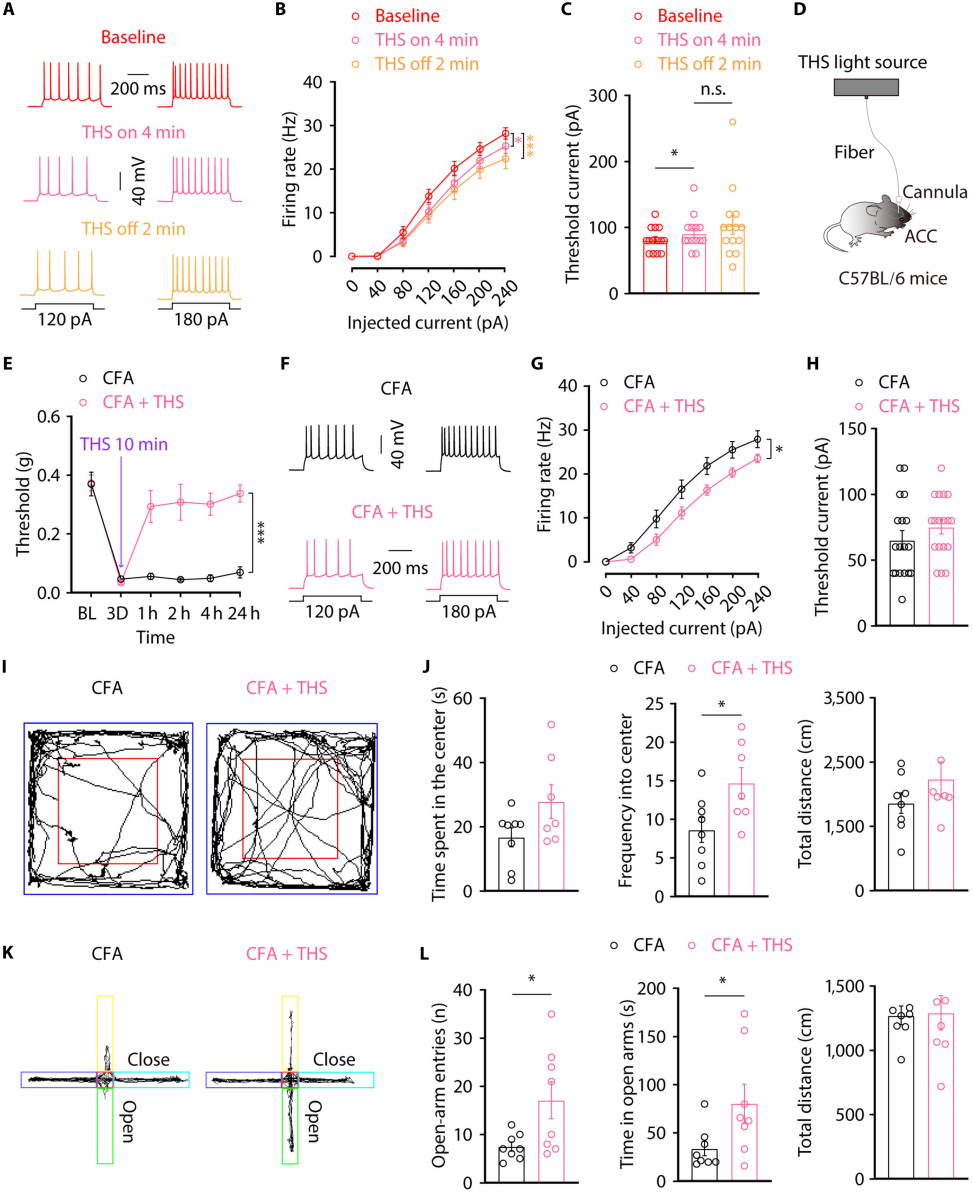
THS-mediated ACC^Glu^ neuronal activity alleviate pain sensitization and anxiety symptoms in CFA mice. (A to C) Representative traces (A) and summarized data of action potentials (B), and rheobase (C) recorded from glutamatergic neurons in the ACC slices from CFA 3D mice before, during, and after THS (*n* = 14 neurons from 3 mice). (D) Schematic diagram of the in vivo THS irradiation in the ACC neurons of CFA 3D mice. (E) Effect of THS irradiation of the ACC brain region on mechanical nociceptive thresholds of CFA 3D mice. Representative traces (F) and summarized data (G) of action potentials recorded from glutamatergic neurons in the ACC slices from CFA 3D mice after THS 24 h (*n* = 18 neurons from 3 mice). (H) Effect of THS on the concentration of glutamate in the ACC, detected by enzyme-linked immunosorbent assay (*n* = 3 mice per group). (I) Representative animal tracks from CFA 3D mice treated with or without THS in the OFT. (J) Performance of CFA 3D mice treated with or without THS in the OFT (*n* = 8 mice per group). (K) Representative animal tracks from CFA 3D mice treated with or without THS in the EPMT. (L) Performance of CFA 3D mice treated with or without THS in the EPMT (*n* = 8 mice per group). Data are shown as means ± SEM. *P < 0.05; **P < 0.01; ***P < 0.001. n.s., not significant. One-way repeated-measures ANOVA with Tukey analysis was used for (B). A 2-tailed paired Student *t* test was used for (C). Two-way repeated-measures ANOVA with Bonferroni post hoc analysis was used for (E) and (G). A 2-tailed unpaired Student *t* test was used for (H), (J), and (L).

Our research has demonstrated that ACC^Glu^ neuronal hyperactivity participate in regulating pain accompanied by anxiety-like behaviors. Therefore, we delved deeper into understanding the functional impact of THS on regulating ACC^Glu^ neuronal excitability in brain slices of CFA 3D mice. We conducted whole-cell recordings from ACC^Glu^ neurons in brain slices of CFA3D mice using THS optical fiber irradiation. When subjected to current injections, we observed a decrease in the spike firing rate (Fig. 5A and B) and an increase in rheobase (Fig. 5C) in ACC^Glu^ neurons during 6 W/cm^2^ THS for 4 min compared to before THS of CFA 3D mice. This inhibitory effect persisted for at least 2 min following cessation of irradiation. These results suggest that THS can effectively modulate electrical signals within ACC^Glu^ neurons.

To investigate whether THS contributes to the inhibition of ACC^Glu^ neurons and alleviates pain accompanied by anxiety-like behaviors, in vivo THS were performed in the ACC of CFA 3D mice. Specifically, we implanted a cannula targeting ACC in mice and inserted infrared fiber into the same area (Fig. 5D). After 10 min of THS on the bilateral ACC of CFA 3D mice, pain threshold testing was conducted. The results showed that THS effectively reversed the pain sensitization behavior induced by CFA, with the effects lasting for a minimum of 24 h (Fig. 5E). Furthermore, we observed that 24 h after THS, the heightened firing of ACC^Glu^ neurons induced by CFA was inhibited (Fig. 5F and G). However, there were no substantial changes in the rheobase of ACC^Glu^ neurons in CFA mice with THS compared to those without THS (Fig. 5H). We then examined changes in anxiety-like behaviors and found that THS significantly increased the frequency of CFA mice entering the center but did not affect the time spent in the central area or distance traveled in the OFT (Fig. 5I and J). Additionally, THS led to a substantial increase in the number of mice entering and the duration of time spent in the open arms but had no effect on the distance traveled in EPMT (Fig. 5K and L). These findings collectively indicate that the suppression of ACC^Glu^ neuron activity by THS helps alleviate the progression of pain and anxiety-like behavior.

### THS reduces the binding ability of gl.utamate to GluA2 subunits of AMPAR

**Fig. 6.**
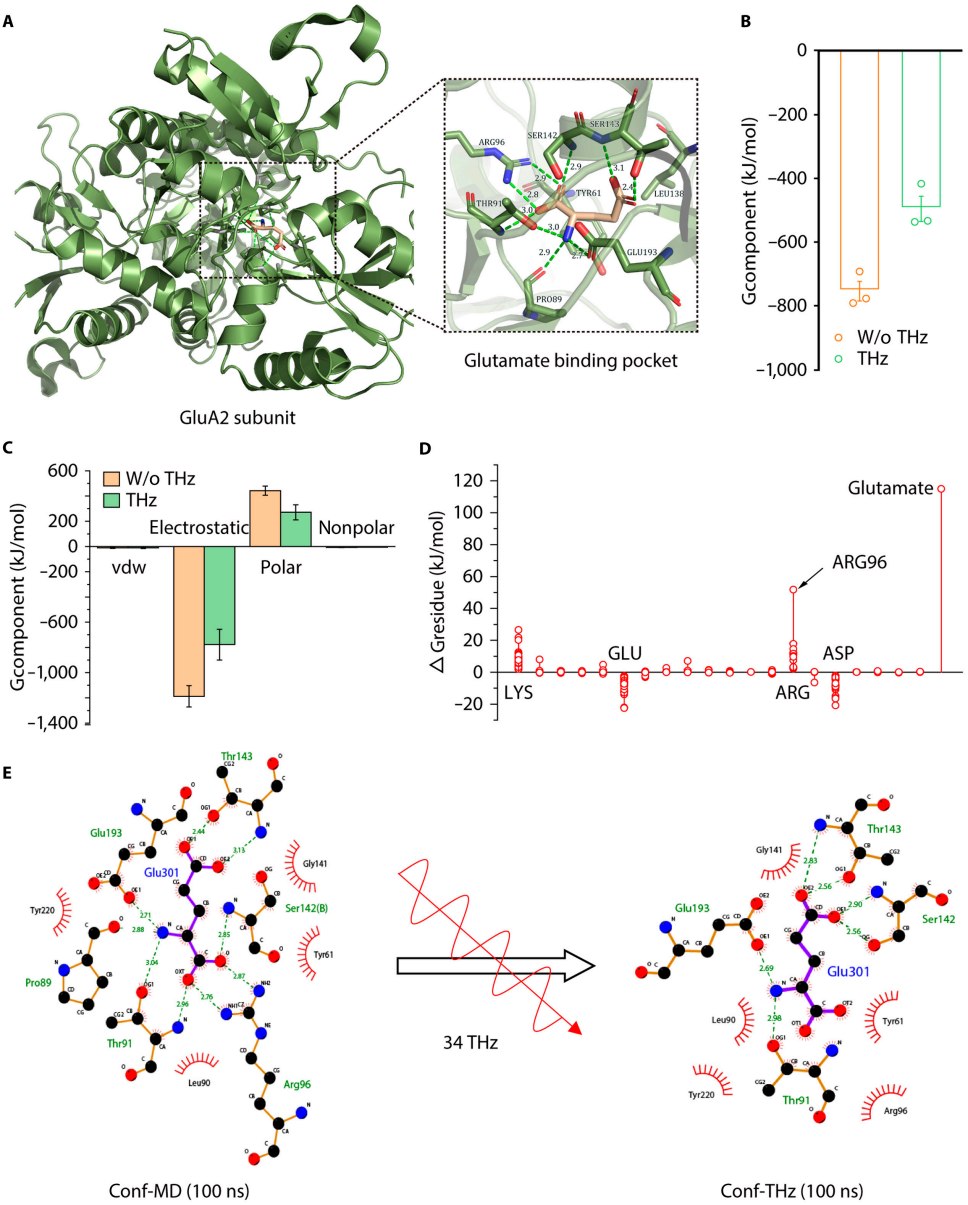
The effects of 34-THz wave on the activity of glutamate on the GluA2. (A) Schematic representation of a GluA2–glutamate complex. The box in the left image depicts the area shown in the right image. The central pink structure represents the glutamate molecule, the green amino acid represents the GluA2 receptor binding pocket with glutamate, the green dotted lines represent hydrogen bonds, and the green background represents the GluA2 subunit of AMPAR. (B) The total binding free energy in the base and 34-THz, 0.6-V/nm irritated case. (C) Four decomposed energetic terms of the total binding free energy between the GluA2 and glutamate in the base case (yellow) and 34-THz, 0.6-V/nm irritated (green) case, respectively. (D) Residue contributions to the variation of the binding free energy after being irritated by a 34-THz, 0.6-V/nm THz wave. All residues are categorized to the type, and each circle denotes one residue. Glutamate represents the glutamate molecule. (E) GluA2–glumate interaction diagram in base case and 34-THz irritated case drawn by LigPlot^+^. The green dashed line represents hydrogen bonding, and the red eyebrows represent hydrophobic interactions. vdw, van der Waals.

Protein–protein interactions are critical and fundamental in many biological processes, typically involving many weak and noncovalent interactions, electrostatic interactions, hydrogen bonds, van der Waals interactions, and hydrophobic effects [41]. Moreover, we can evaluate the binding energy of protein–protein interactions using computational approaches, such as free energy perturbation, thermodynamic integration, molecular mechanics/Poisson–Boltzmann surface area, and molecular mechanics/generalized Born surface area methods [42,43]. In order to gain a deeper understanding of the molecular mechanisms underlying the interactions between THz waves and ACC^Glu^ neurons, we conducted the molecular dynamics simulation of the binding of glutamate and GluA2 subunits. In the GluA2–glutamate complex, 2 hydrogen bonds are formed between glutamate and Arg96 in the glutamate binding pocket in the base case (Fig. 6A). Before carrying out the analysis, we calculated its root mean square deviation to ensure that the system had reached dynamic equilibrium (Fig. S2). The binding free energy between glutamate and GluA2 subunits was calculated with and without THz, and it was found that total binding free energy was about −740 kJ/mol without THz waves (Fig. 6B). Upon decomposing the binding process into individual residues, we found that only the residues in the glutamate binding pocket effectively contributed to the binding in either a negative or positive way (Fig. 2C). Among them, the Arg96 and glutamate contribute mainly through electrostatic attraction (Fig. 2C). To investigate the impact of THz wave on the binding process, we calculated the binding free energy under 34 THz at an amplitude of 0.6 V/nm. Our results show a substantial increase in binding free energy by 35% to −482 kJ/mol (Fig. 6B), which signifies a marked reduction in affinity. We compared the 4 energetic components of binding free energy with and without a 34-THz wave. The decreased affinity can be explained by the increased electrostatic energy caused by the 34-THz wave. (Fig. 6C). After assigning contributions to the binding free energy variances of the specific residues, our analysis highlighted that it was predominantly the amino acids Arg96 that caused a substantial alteration in △*G* (Fig. 6D). The hydrogen bond is essential for maintaining the stability of the interaction between and GluA2 and glutamate. As shown in Fig. 6E, the analysis of the interaction between glutamate and GluA2 indicates that the 34-THz wave can break the hydrogen bond, mainly between the Arg96 and glutamate. Therefore, our simulation indicates that the 34-THz wave can break the hydrogen bond between GluA2 and glutamate and then promote the dissociation of glutamate and GluA2.

## Discussion

In this study, we defined the neuronal activity in ACC, through which antinociception and antianxiety are mediated by THS. Central to this process, ACC^Glu^ neurons are activated during the period of comorbid anxiety-like behavior in pain, and THS acts on the ACC brain region to reduce the binding capacity of glutamate to CluA2 of AMPAR and inhibit the hyperactivity of ACC^Glu^ neurons, thereby alleviating the development of accompanied anxiety in pain. We found that THS, a neuromodulation approach, provides nonthermal and long-term modulation of neuronal activity and behavior.

The ACC is a crucial structure that processes and regulates pain signaling and related feelings. ACC^Glu^ neurons respond to pain stimuli by increasing the frequency of action potential firing [44,45]. In particular, ACC shows hyperactivity in case of chronic pain. This activation causes chronic pain and creates negative emotions, while the inhibition of ACC alleviates the above phenotypes [46,47]. Specifically, we obtained our results by manipulating the ACC^Glu^ neurons with chemical genetics and observed that inhibiting these neurons can relieve pain and related anxiety. In addition, we found that THS could induce antinociceptive and antianxiety effects by calming ACC^Glu^ neurons in CFA mice. Our molecular dynamics simulations showed that 34 THz disrupts the hydrogen bond formed between glutamate and Arg96, reducing the ability of glutamate to bind to the AMPAR and then promoting the dissociation of glutamate and GluA2, which may be one of the reasons for the decrease in excitability of ACC^Glu^ neurons. Interestingly, when the bilateral ACC was irradiated with THz wave for 10 min each on the third day after CFA injection, its antinociceptive effect persisted for at least 24 h. The excitability of ACC^Glu^ neurons ultimately showed a reduced effect after THS, indicating that THS may have a major influence on the regulation of ACC neuron activity. These data suggest that THS has the potential to inhibit the progression of chronic pain and comorbid anxiety after inflammation. Evidence supporting this possibility has been proved through the clinical application of photobiomodulation therapy, which effectively promoted pain relief in patients receiving hip arthroplasty [48]. However, it is not yet known how it works.

Numerous studies have explored the biological effects of infrared light stimulation at near-infrared wavelengths, particularly those with high absorption by water, such as infrared light activates CNS neurons largely due to a transient increase in local temperature [49–51]. The THz wave attenuation by ACSF indicates a low attenuation in solution at the wavelength used in the present study. Under our experimental conditions, THS still caused an increase in temperature but less than 1 °C near the fiber tip (<50 μm), suggesting that THS exerts nonthermal effects on neuronal signaling to the distance of 300 μm or more from the fiber tip. Unlike optogenetic manipulation [52], THS requires no exogeneous gene expression, making it more suitable for clinical subjects. Broadband, high-responsivity optoelectronic applications show great potential [53,54]. These accumulated evidence suggest that THS has great potential as a readily available and side-effect-free additional option for analgesic and antianxiety. Recent studies have shown that 36 THz decreases the excitability of pyramidal neurons in the ACC by increasing the conductivity of voltage-gated potassium ion channel, resulting in the relief of neuropathic pain in mice [55]. Although our study demonstrated the regulatory effect of THS with a specific wavelength on neuronal activity, analgesic, and antianxiety, it cannot be ruled out that other wavelengths may have similar effects, and additional research is required to identify the optimal regulatory wavelength. The ACC^Glu^ neurons exhibit numerous axonal projections in various regions such as nucleus accumbens, ventral tegmental area, thalamus, and other areas. Whether these projections and molecular mechanism are associated with THS induced analgesia and antianxiety and the mechanisms responsible for the changes in neuronal activity caused by THS are intriguing questions that require further investigation.

Taken together, we exploit the regulatory effect of THS on the nervous system to establish a novel approach for relieving pain and anxiety. This approach could accelerate research on light-induced analgesia and antianxiety, with the goal of addressing the current challenges of prolonged treatment duration, limited efficacy, and adverse drug reactions. Also, our study provides a possible strategy and target for nonpharmacological interventions in the future, particularly in the management of chronic pain and associated emotional disturbances.

## Materials and Methods

### Animals

All animal procedures were approved by the Institutional Animal Care and Use Committee of the University of Electronic Science and Technology of China. Male C57BL/6J mice were used and were obtained from Spearfish (Beijing) Biotechnology Co., China. Mice were used in all experimental studies were 8 to 10 weeks old and housed 3 to 5 per cage. The mice have free access to food and water in the cages. The temperature of the mice’s living environment was controlled between 23 and 25 °C, and 12 h of light and 12 h of darkness were maintained.

### Animal model of inflammatory pain

We anesthetized the mice using isoflurane and injected 20 μl of CFA (Sigma-Aldrich) into the metatarsal surface of the left hind paw of all mice using an insulin injection needle. The same volume of 0.9% saline was injected into the left hind paw of the control group.

### von Frey filament test

We placed the mice on a wire mesh and isolated each mouse in a separate space with a breathable and transparent plastic box and allowed them to acclimate to the environment for 30 to 40 min before testing the mice’s mechanical withdrawal threshold using calibrated von Frey’s filament. During the test, the pressure of the von Frey filament was varied from small to large, and the value of the pressure of the von Frey filament at that point was recorded when the mice retracted or licked their paws, which was the mechanical withdrawal threshold of the mice. Three positive responses were recorded for each mouse, and the mean value was calculated. Throughout the experiment, the experimenters remained unaware of the group identity.

### THz sources

A pulsed QCL from Daylight Solutions Inc. was used to control the wavelength and power of a laser. This laser emits infrared radiation in the range of 5 to 11 μm, and its output was connected to a 400-μm diameter infrared fiber (numerical aperture: 0.30 ± 0.03). During in vitro brain slice irradiation, an infrared fiber was affixed to the robotic arm of a micromanipulator (Sutter Instruments) and moved through said micromanipulator to position itself above the brain slice near the ACC. At this point, a glass electrode was manipulated using another micromanipulator to clamp onto ACC neurons for electrophysiological recording. In the stimulation phase, the recorded cell was exposed to a sequence of THZ waves with a frequency of 34 THz, a repetition rate of 200 kHz, and a pulse width of 500 ns; the power density at the end of the laser fiber in air was approximately 1 or 6 W/cm^2^.

### In vivo THz irradiation

We first inserted a cannula (with an internal diameter of 0.34 mm, RWD) into the ACC of a mouse that was under anesthesia and immobilized in a stereotactic frame. To conduct in vivo THZ wave tagging of ACC, mice were anesthetized with 1.5% isoflurane gas at a flow rate of 0.5 and secured in place using a stereotaxic frame for in vivo irradiation. The catheter cap was removed, and the 240-μm infrared fiber was inserted into the embedded catheter, accurately positioning it to the ACC. The frequency and power of infrared fiber were controlled using a QCLpoint frequency laser. Each side was irradiated for 10 min. Pain behavioral testing was conducted after half an hour of THz wave exposure, and anxiety-like behavior testing was conducted after 24 h. Mice were given a minimum of 10 d to recover before THz irradiation to reduce stress during pain testing. Mice that did not have precise injection sites were not included in the study.

### Molecular dynamics simulations

The ligand-binding domain was initially placed in the center of a cubic simulation box with a side length of 87Å. The simulation box contained 19,154 water molecules (Tip3 model), 54 potassium ions, and 63 chloride ions (physiological concentration: 0.15 M) to preserve electrical neutrality of the entire simulation system. The solvated protein–ligand complex system was created by GROMACS software with charmm-36 force field and then equilibrated for 10.625 ns at a temperature of 303.15 K and a pressure of 1 bar. During the molecular dynamics simulations, the protein and ligand undergo position restraints with gradually decreasing force constants until reaching zero, so the protein–ligand complex system is sufficiently relaxed. In the baseline case without THz wave irritation, the system is simulated for 100 ns with trajectories recorded per 10 ps. In the THz wave irritation situation, a 100-ns THz wave was introduced to the fully equilibrated system. In the repeatability test, the corresponding simulation case was simply repeated for 3 more times without any parameters changed and the trajectories were recorded per 10 ps.

The simulation system consisted a GluA2–glutamate complex, solvated in a 0.15 M NaCl solution. The structure of GluA2–glutamate complex came from the Research Collaboratory for Structural Bioinformatics protein databank (ID: 4O3B). The molecular dynamics simulations were conducted using the GROMACS 2021.3 withcharmm36 force fields utilized to accurately depict the GluA2–glutamate complex. The dynamics of the GluA2 and glutamate molecules before and after THz irradiation, as well as the kinetic processes involved in ligand-receptor binding, are at the nanoscale.

The system had been well balanced at a temperature of 303.15 K and a pressure of 1 bar, the process of balancing has been described in detail in our previous research [7]. After the simulation system achieved full equilibrium, in the base case without external THz, the trajectories of the atoms were recorded for up to 100 ns for subsequent analysis. In the case of irritation, an electric field with an intensity of 0.6 V/nm was introduced to the simulation system. The electric field acts the desired electromagnetic field, whereas the impact of the magnetic component is insignificant. Importantly, repeat test were conducted for the base and irritated cases to validate the conclusions.

In the calculation of the binding free energy (*G*_binding_), there are 4 parts need to consider, namely van der Waals free energy (*E*_vdw_), electrostatic free energy (*E*_elec_), polar solvation energy (*E*_polar_), and nonpolar solvation energy (*E*_nonpolar_) [56]. The g_mmpbsa tool was utilized to compute the binding free energy between the GluA2 and glutamate. The trajectory snapshots were sampled every 1.0 ns ensuring so that they were independent and suitable for calculating average binding free energies using the bootstrap method. We obtained the hbond change between the base case and the irritated case via GROMACS’ Hbond analysis. The H-bond network and hydrophobic between glutamate receptor and glutamate was displayed by Ligplot^+^ software.

### Whole-cell patch-clamp recordings

We followed established protocols to prepare acute brain slices [57]. Mice were deeply anesthetized with pentobarbital sodium and perfused with standard ACSF solution. Coronal slices containing the ACC were cut at 300-μm thickness and incubated in ACSF. For whole-cell recording, slices were transferred to a slice chamber and perfused with standard ACSF solution.

We used a vertical microscope (Olympus) equipped with a 40× water immersion objective and an infrared camera to visualize ACC neurons on a video monitor and recorded them with whole-cell patch clamp. The signals were sampled at a rate of 10 kHz, filtered with a low-pass filter at 2.8 kHz, and recorded using a Multiclamp 700B amplifier. Data were gathered from neurons that had an input resistance exceeding 100 MΩ and a series resistance below 30 MΩ. If the series resistance fluctuated by more than 20% during the recording, the experimental session was promptly halted. The minimum current required to initiate an action potential was identified as the threshold current.

### Temperature measurement

We utilized an in-line temperature sensor from the automatic thermostat (Warner Instruments) to monitor the temperature variations of the standard ACSF with THz irradiation. The distance between the temperature sensor and the optical fiber is adjusted through a micromanipulator.

### Open-field test

The open field of mice is an area of 50 cm × 50 cm × 60 cm, which includes a 25 cm × 25 cm square area. At the start of the experiment, the mice are placed in the center of the field to freely explore. The Smart v3.0 software is used to track their movement trajectory for a total of 5 min, analyzing the number of times they enter the central area and the time they spend in the central area. After each test, the instrument was cleaned with 75% ethanol to eliminate any olfactory cues.

### Elevated plus maze test

The elevated maze is composed of 4 identical plastic arms of 6 × 60 cm, presenting a cross shape. Two of the opposing arms are enclosed by a 30-cm-high plastic wall, while the other 2 arms are open. Mice are placed on a central platform at the start of the experiment, facing the open arm. The Smart v3.0 software is used to track their movement trajectory for a total of 5 min, analyzing the time the mice spend opening their arms and the number of times they enter the open arms. The maze is cleaned with 75% ethanol after each test to remove any olfactory cues.

### Chemogenetic manipulation

Mice were anesthetized with intraperitoneal injection of pentobarbital (20 mg/kg) and fixed on a stereotaxic device. An infusion pump was used to inject the virus into ACC at a rate of 30 nl/min. To avoid virus overflow, the injection needle was left for 5 to 10 min after injection was completed and the needle was removed. The scalp was sutured using a suture needle and thread and disinfected with iodophor. The coordinates were specified as the distance from the brain surface in the dorsal ventral (DV) direction, the distance from the midline in the medial lateral (ML) direction, and the distance from the bregma in the anterior posterior direction (in millimeters).

To manipulate chemogenetics, the 150-nl rAAV-CaMKIIa-hM4Di-mCherry-WPRE-pA virus (AAV-CaMKIIa-hM4Di-mCherry, AAV2/9, 3.69 × 10^13^ vg/ml) was injected into the ACC (AP, +0.50 mm; ML, −0.25 mm; DV, −1.08 mm) of C57BL/6J mice at coordinates AP +0.50 mm, ML −0.25 mm, and DV −1.08 mm. Three weeks later, the mice received an intraperitoneal injection of 5 mg/kg CNO (MedChemExpress) 30 min before behavioral tests. The rAAV-Ef1α-DIO-mCherry-WPRE-pA virus (AAV-DIO-mCherry, AAV2/8, 8.93 × 10^12^ vg/ml) was used as a control. Following behavioral testing, mice were transcardially perfused with precooled 0.9% saline and 4% paraformaldehyde. Mice with missed injections were excluded from the study.

### Statistical analysis and drugs

Electrophysiological recordings data were analyzed offline using Clampfit software version 10.6. Statistical analyses and graphing were performed using GraphPad Prism 9 and OriginPro 2018 software. Statistical comparisons between 2 groups were performed using paired or unpaired Student *t* tests. For analyses with multiple experimental groups, 1-way and 2-way analysis of variance (ANOVA) and Bonferroni post hoc analyses were utilized. Animals were randomly assigned to experimental groups to minimize the impact of other variables on the results. Data are presented as individual values or as the mean ± standard error of the mean (SEM), with significance levels denoted as *P < 0.05, **P < 0.01, and ***P < 0.001, and not significant (n.s.). *P* values less than 0.0001 are not provided as exact values. All number of individual experiments (*n*), significance analyses, statistical tests, and other pertinent information for data comparison are detailed in Table S1. Unless specified otherwise, all drugs were obtained from Sigma-Aldrich.

## Data Availability

The data that support the findings of this study are available from the corresponding author upon reasonable request.
