# Supplementary material for: Terahertz Wave Alleviates Comorbidity Anxiety in Pain by Reducing the Binding Capacity of Nanostructured Glutamate Molecules to GluA2
Source: Research (Wash D C). 2024 Dec 11;7:0535. doi: 10.34133/research.0535 (PMC11633831; doi:10.34133/research.0535)
Supplement: Supplementary 1 — Figs. S1 and S2 Table S1 [file research.0535.f1.pdf]

Supplementary Figure

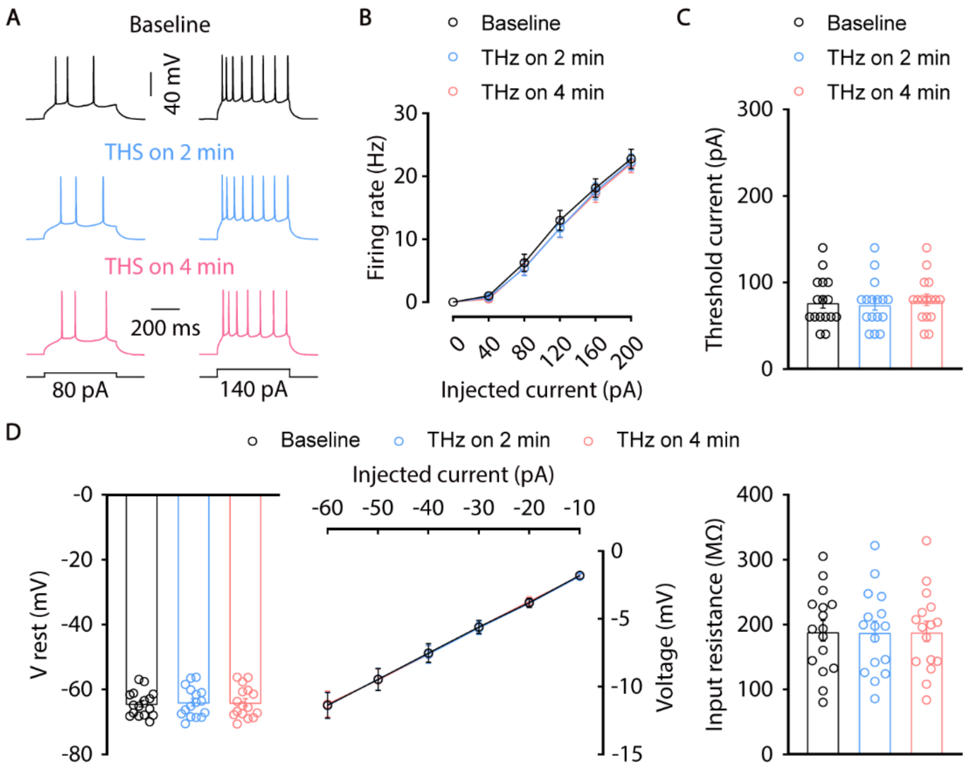

Supplementary Fig. 1. 1 W/cm^2 34 THz wave has no significant effect on the excitability of ACC^{Glu} neurons. Representative traces (A) and summarized data (B) of action potentials; rheobase (C), resting membrane potential, voltage-current plots of voltage responses to a stepwise series of hyperpolarizing currents (-10 to -60 pA, -10 pA/step; duration: 500 ms), and input resistance (D) recorded from glutamatergic neurons in the ACC slices from mice before and during THS (n = 16 neurons from three mice). Data are shown as means ± SEM. One-way RM ANOVA with Tukey analysis was used for B, C, D.

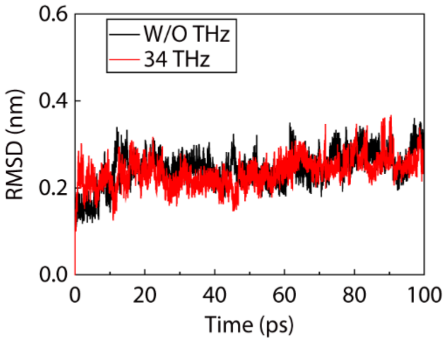

Supplementary Fig. 2. The time-varying curve of RMSD. Achieving dynamic equilibrium in systems with and without 34 THz.

Table S1. Statistical analyses related to figures 2-5 and supplementary figure 2.

Figure	Conditions (sample size)		Analysis	P value	t or F value
2H			One-way RM ANOVA with Tukey analysis	$P = 0.0070$	F (1.133, 6.801) = 13.84
	Baseline (14)	THz on 2 min (14)		$P = 0.0473$	
	Baseline (14)	THz on 4 min (14)		$P = 0.0343$	
	Baseline (14)	THz off 2 min (14)		$P = 0.0329$	
	THz on 4 min (14)	THz off 2 min (14)		$P = 0.1571$	
2I	Threshold current				
	Baseline (14)	THz on 2 min (14)	Paired two sample t test	$P = 0.0011$	t (13) = 4.163
	Baseline (14)	THz on 4 min (14)	Paired two sample t test	$P = 0.0038$	t (13) = 3.513
	THz on 4 min (14)	THz off 2 min (14)	Paired two sample t test	$P = 0.4533$	t (13) = 0.7731
	V rest				
	Baseline (14)	THz on 2 min (14)	Paired two sample t test	$P = 0.0081$	t (13) = 3.119
	Baseline (14)	THz on 4 min (14)	Paired two sample t test	$P = 0.0029$	t (13) = 3.660
	THz on 4 min (14)	THz off 2 min (14)	Paired two sample t test	$P = 0.1437$	t (13) = 1.556
	Input resistance				
	Baseline (14)	THz on 2 min (14)	Paired two sample t test	$P = 0.4634$	t (13) = 0.7554
	Baseline (14)	THz on 4 min (14)	Paired two sample t test	$P = 0.6081$	t (13) = 0.5255
	THz on 4 min (14)	THz off 2 min (14)	Paired two sample t test	$P = 0.2698$	t (13) = 1.153
3B	Saline (8)	CFA (8)	Two-way RM ANOVA with Bonferroni post hoc analysis	$P < 0.0001$	F (1, 14) = 134.8
	BL			$P > 0.9999$	
	1 Day			$P = 0.0003$	
	3 Day			$P < 0.0001$	
	5 Day			$P = 0.0004$	
	7 Day			$P = 0.0370$	
	10 Day			$P = 0.0371$	
	14 Day			$P > 0.9999$	
3D	Saline (22)	CFA 3D (21)	Two-way RM ANOVA with Bonferroni post hoc analysis	$P = 0.0005$	F (1, 41) = 14.44

3F	Saline (7)	CFA 3D (7)	Unpaired two sample t test		
	Time spent in the center			*P* = 0.0392	t (12) = 2.315
	Frequency into center			*P* = 0.2860	t (12) = 1.117
	Total distance			*P* = 0.1033	t (12) = 1.763
3H	Saline (8)	CFA 3D (8)	Unpaired two sample t test		
	Open arm entries			*P* = 0.0017	t (14) = 3.859
	Time in open arms			*P* = 0.0012	t (14) = 4.032
	Total distance			*P* = 0.1780	t (14) = 1.418
4D	mCherry (8)	hM4Di-mCherry (8)	Two-way RM ANOVA with Bonferroni post hoc analysis	*P* < 0.0001	F (1, 14) = 131.9
	BL			*P* > 0.9999	
	CFA			*P* > 0.9999	
	0.5 h			*P* = 0.0042	
	1 h			*P* = 0.0001	
	1.5 h			*P* =0.0010	
	2 h			*P* = 0.0003	
	2.5 h			*P* = 0.0002	
	3 h			*P* < 0.0001	
	3.5 h			*P* > 0.9999	
4F	mCherry (8)	hM4Di-mCherry (7)	Unpaired two sample t test		
	Frequency into center			*P* = 0.0354	t (13) = 2.347
	Time spent in the center			*P* = 0.0090	t (13) = 3.065
	Total distance			*P* = 0.1207	t (13) = 1.661
4H	mCherry (7)	hM4Di-mCherry (7)	Unpaired two sample t test		
	Open arm entries			*P* = 0.0076	t (12) = 3.200
	Time in open arms			*P* = 0.0137	t (14) = 2.887
	Total distance			*P* = 0.6185	t (12) = 0.5111
5B			One-way RM ANOVA with Tukey analysis	*P* = 0.0123	F (1.116, 6.697) = 11.07
	Baseline (14)	THz on 4 min (14)		*P* = 0.0261	
	Baseline (14)	THz off 2 min (14)		*P* = 0.0002	
	THz on 4 min (14)	THz off 2 min (14)		*P* = 0.1106	
5C	Baseline (14)	THz on 4 min (14)	paired two sample t test	*P* = 0.0475	t (13) = 2.188
	Baseline (14)	THz off 2 min (14)	paired two sample t test	*P* = 0.0438	t (13) = 1.847
	THz on 4 min (14)	THz off 2 min (14)	paired two sample t test	*P* = 0.2196	t (13) = 1.290

5E	CFA (9)	CFA + THS (9)	Two-way RM ANOVA with Bonferroni post hoc analysis	*P* < 0.0001	F (1, 16) = 88.60
5G	CFA (18)	CFA + THS (18)	Two-way RM ANOVA with Bonferroni post hoc analysis	*P* = 0.0208	F (1, 34) = 5.878
5H	CFA (18)	CFA + THS (18)	Unpaired two sample t test	*P* = 0.2695	t (34) = 1.122
5J	CFA (8)	CFA + THS (7)	Unpaired two sample t test		
	Time spent in the center			*P* = 0.0808	t (13) = 1.893
	Frequency into center			*P* = 0.0323	t (13) = 2.397
	Total distance			*P* = 0.2430	t (13) = 1.223
5L	CFA (8)	CFA + THS (8)	Unpaired two sample t test		
	Open arm entries			*P* = 0.0285	t (14) = 2.441
	Time in open arms			*P* = 0.0443	t (14) = 2.210
	Total distance			*P* = 0.8920	t (14) = 0.1383
S1B			One-way RM ANOVA with Tukey analysis	*P* = 0.0098	F (1.301, 6.506) = 11.79
	Baseline (16)	THz on 2 min (16)		*P* = 0.0541	
	Baseline (16)	THz on 4 min (16)		*P* = 0.0198	
	THz on 2 min (16)	THz on 4 min (16)		*P* = 0.3350	
S1C	Threshold current		One-way RM ANOVA with Tukey analysis	*P* = 0.1884	F (1.731, 25.97) = 1.800
	Baseline (16)	THz on 2 min (16)		*P* = 0.5881	
	Baseline (16)	THz on 4 min (16)		*P* = 0.7042	
	THz on 2 min (16)	THz on 4 min (16)		*P* = 0.0972	
S1D	V rest		One-way RM ANOVA with Tukey analysis	*P* = 0.2750	F (1.217, 18.25) = 1.318
	Baseline (16)	THz on 2 min (16)		*P* = 0.3988	
	Baseline (16)	THz on 4 min (16)		*P* = 0.5458	
	THz on 2 min (16)	THz on 4 min (16)		*P* = 0.9665	
	Voltage		One-way RM ANOVA with Tukey analysis	*P* = 0.2652	F (1.499, 7.497) = 1.554
	Baseline (16)	THz on 2 min (16)		*P* = 0.1299	
	Baseline (16)	THz on 4 min (16)		*P* = 0.9931	
	THz on 2 min (16)	THz on 4 min (16)		*P* = 0.4316	
	Input resistance		One-way RM ANOVA with Tukey analysis	*P* = 0.9319	F (1.556, 23.34) = 0.03785
	Baseline (16)	THz on 2 min (16)		*P* = 0.9657	
	Baseline (16)	THz on 4 min (16)		*P* = 0.9843	
	THz on 2 min (16)	THz on 4 min (16)		*P* = 0.9958	
